# An Internet Snapshot Survey Assessing the sale of Synthetic Cannabinoid Receptor Agonists for use with Electronic Vaping Devices

**DOI:** 10.1007/s13181-024-01013-0

**Published:** 2024-06-05

**Authors:** Allon Gould, Paul I Dargan, David M Wood

**Affiliations:** 1 Respiratory Medicine, Whipps Cross University Hospital, Barts Health NHS Trust, London, UK; 2 Clinical Toxicology, Guy’s and St Thomas’ NHS Foundation Trust, London, UK; 3 Clinical Toxicology, Faculty of Life Sciences and Medicine, King’s College London, London, UK

**Keywords:** Synthetic cannabinoids, Vape, Electronic vaping devices, Novel psychoactive substances, Internet snapshot survey

## Abstract

**Introduction:**

Synthetic cannabinoid receptor agonists (SCRAs) are associated with significant toxicity and are increasingly used in electronic vaping devices. We assessed the availability of SCRA vaping products to UK purchasers on the surface web.

**Methods:**

An internet snapshot survey was performed between October 2022 and January 2023 on ‘google.com’ using the search terms “buy c-liquid vape”, “buy herbal incense vape liquid”, “buy cannabis vape liquid”, “buy hashish vape liquid”, “buy K2 vape liquid”.

**Results:**

62 websites selling 128 SCRA vaping brands were identified. Most were purportedly based in the USA (41 websites, 66%) and most sold other controlled substances. Purchase incentives offered included discreet packaging (38, 61%), discounts for bulk purchase (34, 55%) and tracked delivery (30, 48%). Many websites stated SCRA products were: not for human consumption (41, 66%), for research purposes only (15, 24%), or legal (28, 45%). Websites sold a median (IQR) of 16 (7–25) SCRA vaping brands. Almost all were bottles of vaping liquid (1220/1225, 99.6%). The most common bottle size was 5mL (60%), the median (IQR) total volume of SCRA liquid per sale was 50mL (10–200mL). Median (IQR) price was £3.39/mL (£2.01/mL– £5.29/mL). Price decreased with increasing volume purchased (£6.58/mL for ≤ 5mL, £1.60/mL for > 200mL).

**Conclusion:**

SCRA vaping products are easily obtainable online, in both small and bulk quantities. Information provided to purchasers on safety and legality is lacking or misleading. Further studies are needed to confirm the chemistry of these products. Policymakers should consider steps to limit the potential harm caused by the purchase and use of these products.

## Introduction

Synthetic cannabinoid receptor agonists (SCRAs) are a structurally diverse group of new psychoactive substances (NPS) that act through high-affinity agonism of cannabinoid receptors [1, 2]. Their psychoactive effects resemble those of cannabis, however they tend to be more potent with longer-lasting effects. Their use has been associated with significant acute toxicity, fatalities, and outbreaks of mass poisonings [3–5].

Since their appearance as NPS over the last two decades, there has been a proliferation in the diversity of SCRAs available. The EMCDDA (European Monitoring Centre for Drugs and Drug Addiction) currently monitors 245 SCRAs with multiple new compounds being detected every year [6].

In the UK, supply, production, importation and exportation of all SCRAs is now illegal, either through specific naming or generic definitions under the 1971 Misuse of Drugs Act or, for those not captured by generic definitions, through the 2016 Psychoactive Substances Act. This has, however, had little effect on rates of SCRA-related deaths [4, 7] or hospital admissions [8, 9] and SCRAs continue to be among the most common substances implicated in UK hospital presentations with acute drug toxicity [10]. Available data suggest that the prevalence of SCRA use is relatively low in the UK, with an estimated 2.7% of 16–59 year olds having ever used any NPS [11]. However, there is higher use amongst certain groups, such as people experiencing homelessness and people who are incarcerated [3]. In a recent survey of people in a UK prison, 46.7% of participants reported use of SCRAs, with 75.9% using SCRAs via e-cigarette [12]. There are also particular concerns about use amongst teenagers and young adults [13, 14].

Despite legislation in the UK and many other countries making the sale of SCRAs illegal, there is evidence of an online SCRA market mediated by websites on both the “surface web” (Internet content accessible through standard search engines) and the “dark web” (intentionally concealed content which is not indexed by traditional search engines; data are generally encrypted, anonymised and require specialist software to access) [15, 16].

The last two decades have also seen a dramatic increase in the use of electronic vaping devices (EVDs) for nicotine use [17], particularly amongst teenagers and young adults [18]. These devices use a heated coil to vaporise an ‘e-liquid’ containing solvent, flavourings and/or active ingredients into an aerosol that is inhaled by the user [19]. Use of EVDs for consumption of unregulated drugs is increasing [20–23]– a practice which can increase ease of consumption and help avoid detection [24]. There is evidence that SCRAs which, until recently, had typically been consumed as smoking mixtures, are increasingly available as preparations for use via EVDs [20, 25, 26].

Although of importance in understanding the UK’s evolving drugs economy and in informing both public health and drug control policy, data on the availability of SCRA vaping products are lacking.

The aim of this study was to assess the availability of SCRA vaping products to UK purchasers on the “surface web” [27], using an Internet Snapshot Survey [28, 29]– a methodology developed by the EMCDDA to standardise monitoring of Internet sites selling drugs.

## Methods

We performed an Internet Snapshot Survey of websites selling SCRA vaping products, with all results being collected between October 2022 and January 2023. Searches were performed on “google.com” using “Google Chrome” browser while signed out of a Google account, from a computer with a UK-based Internet Protocol (IP) number. Prior to each search being performed, browsing history, download history, cookies and cached data were cleared, and “make searches and browsing better” and “FLoC” (Federated Learning of Cohorts) were turned off.

Potential search terms were generated by the authors– clinical toxicologists with expertise in new psychoactive substances– and by a preliminary survey of websites selling SCRA products. Trial searches were then performed, in order to identify terms that provided a comprehensive set of websites selling SCRA vape products, while minimising irrelevant websites. The final search terms chosen were: “buy c-liquid vape”, “buy herbal incense vape liquid”, “buy cannabis vape liquid”, “buy hashish vape liquid” and “buy K2 vape liquid”.

As per EMCDDA methodology [28, 29], for each search term, the first 100 Internet sites identified were reviewed in full, and every further search result was then reviewed in full until 20 successive irrelevant websites were found.

Websites were excluded if they were unavailable or unresponsive, did not sell or were out of stock of SCRA vaping products, did not ship to the UK, were linked to a vendor but did not directly sell products, or if it was not possible to proceed to checkout. Each unique website was only included once in the final analysis.

Included websites were reviewed in greater detail to extract the following information: country of origin of website– determined from listed address, national dialling code of contact phone number, statements of country of origin on the website, or nation-specific domain name suffix; countries supplied to– determined from statements on the websites and at checkout; country of production– determined from statements on each website. Payment options were reviewed at checkout. Product catalogues were reviewed in full for other drugs and paraphernalia on sale. Websites were reviewed for any statement that one or more of the SCRA vaping products were legal, not for human consumption or for research purposes only. Incentives or discounts mentioned anywhere on the website were documented.

SCRA vaping products were defined as any SCRA compound or brand being sold in a preparation primarily intended for use via EVD. SCRA sprays, powders and infused papers were not included. Refined cannabis extracts including cannabidiol (CBD), tetrahydrocannabinol (THC), cannabigerol (CBG) and hexahydrocannabinol (HHC) were not included. Detailed data were collected about each SCRA vaping product on sale, including compound name if listed, form of product, volume on sale and price. Where products had different prices for different volumes of the product, these were each listed. Prices were converted to GBP (British pound) using date-specific exchange rates as per the European Central Bank website [30].

### Statistical Analysis

Percentages and proportions were used to describe the results. Where data distributions were described, median, interquartile ranges (IQR) and ranges were used as data were skewed (Shapiro Wilks test for number of vaping products per website: *p* = 0.001, for number of payment options per website: *p* < 0.001, for all five price categories: *p* < 0.001).

## Results

### Websites

Of 612 search results, we identified 62 unique websites selling SCRA vaping products. The majority of websites selling SCRA vaping products were based in the USA (41, 66%), followed by the UK (9, 15%), Republic of Ireland (2, 3%), Australia (1, 2%), The Netherlands (1, 2%) and Norway (1, 2%). No country of origin could be found for 7 (11%) websites.

Of the 62 websites selling SCRA vaping products, 42 (68%) reported supplying products worldwide, while 13 (21%) did not report countries supplied to. The remaining 20 websites supplied to a combination of the UK (6), “Europe” (6), the USA (5), “Asia” (3), Canada (3), Australia (3) and the United Arab Emirates (2). One website explicitly did not supply to the USA.

Most websites selling SCRA vaping products (42, 68%) did not state where their products were supplied from. Where listed, countries supplied from included the USA (9), the UK (5), Australia (3), Canada (3), “Europe” (3), Sweden (3), The Netherlands (3), The Dominican Republic (1), Mexico (1), Spain (1) and Portugal (1). Most websites (55, 88%) did not state the country of manufacture of their products; where this was listed, countries included UK (3), USA (3), Canada (3), Australia (2), New Zealand (1), Vietnam (1) and Spain (1).

A minority of websites exclusively sold SCRA vaping products (3, 5%). Other products sold by websites included: other SCRA preparations (57, 92%), cannabis or THC products (42, 68%), non-NPS illegal recreational drugs (including heroin, cocaine, MDMA (3,4-methylenedioxymethamphetamine), amphetamine, methamphetamine, ketamine, LSD (lysergic acid diethylamide) and hallucinogenic mushrooms) (34, 55%), CBD products (32, 51%), other NPS (27, 44%), vaping paraphernalia (19, 31%) and nicotine products (5, 8%).

A variety of electronic payment options were available for purchasing SCRA vaping products. Most websites offered multiple payment method options (median: 3, IQR 2–4, Range: 1–9). 57 (92%) websites offered payment with cryptocurrency, 42 (68%) by payment app or digital wallet, 19 (31%) by bank or wire transfer, 9 (15%) by debit or credit card payment, and 9 (15%) with digital gift card.

Websites selling SCRA vaping products offered a variety of incentives for purchasing their products (Table 1). The most common were: discreet packaging (38, 61%), discounts for bulk purchase (34, 55%), tracked delivery (30, 48%) and same-day shipping (28, 45%). 41 (66%) websites stated some or all of their products were not for human consumption, 15 (24%) stated some or all of their products were for research purposes only, and 28 (45%) stated some or all of their products were legal.

**Table 1:** Incentives offered to potential purchasers (by 62 websites selling SCRA vaping products)

Incentives	Number (%) of websites
Discreet packaging	38 (61%)
Discount for bulk purchase	34 (55%)
Tracked delivery	30 (48%)
Same-day shipping	28 (45%)
Product purity guarantee / quality-controlled	27 (44%)
Free shipping	24 (39%)
Refunds possible	19 (31%)
Specialist packaging to prevent damage to product / evade customs	17 (27%)
Returns possible	15 (24%)
Reshipping for lost packages	14 (23%)
Discount for returning customers	9 (15%)
Discount for using specific payment method	7 (11%)
Guarantee no long-term buyer records kept	6 (10%)
Multiple flavours	6 (10%)
Discreet billing / encrypted orders	4 (6%)
Unbranded product	4 (6%)
Discount for new customers	4 (6%)
Free samples	3 (5%)
Gift with purchase	2 (3%)
Discount coupons available	2 (3%)
Price match guarantee	2 (3%)
Fake return address	1 (2%)
Discount for “medical use”	1 (2%)
Discreet packaging for delivery to prisons	1 (2%)

## Products

128 unique SCRA vaping product brands were identified across websites. Websites sold a median of 16 individual SCRA vaping product brands (IQR: 7–25; range: 1–58). Table 2 shows the most common SCRA vaping product brands on sale.

**Table 2:** SCRA vaping product brands available (from 62 websites selling SCRA vaping products)

SCRA vaping product brand	Number (%) of websites selling brand
Bizarro	45 (73%)
Diablo	37 (60%)
Blazing Blueberry	36 (58%)
Kush	34 (55%)
Aloha Tangerine	33 (53%)
K2 Code Red	33 (53%)
Mr Nice Guy	32 (52%)
Cloud 9	32 (52%)
AK-47 Adios	30 (48%)
7 H Hawaiian punch	28 (45%)
Klimax Berry Colada	28 (45%)
Joker	28 (45%)
Angry birds	27 (44%)
Buzz	25 (40%)
Mr Nice Guy Chronic Hypnotic	23 (37%)
Black label	22 (35%)
xXx Strawberry Splash	21 (34%)
5 F-AKB-48 c-liquid	20 (32%)
Cannabinoid C-Liquid	20 (32%)
White Tiger	18 (29%)
Mad Hatter Sour Gummy	18 (29%)
Green Giant	18 (29%)
Purple Blossom	18 (29%)
Brain Freeze	17 (27%)
Kratom Meang Da	17 (27%)
Pink Blossom	16 (26%)
Mind Trip	16 (26%)
Code 69	16 (26%)

Of 1225 individual SCRA vaping product listings, only 58 (4.7%) had an SCRA compound named, with another 46 (3.8%) stating they contained a “synthetic cannabinoid”. The most commonly listed SCRA compound was 5 F-AKB48 (36 listings, 2.9%). Other compounds listed were: 5 F-MDMB-2201 (5, 0.4%), 5 F-MDMB-PINACA (5, 0.4%), AB-CHFUPYCA (3, 0.2%), 5 F-ADB (2, 0.2%), 5 F-CUMYL-PINACA (2, 0.2%), JWH-018 (2, 0.2%), SGT-25 (2, 0.2%) and Hu-210 (1, 0.1%).

The vast majority of products were sold as bottles of liquid for refilling EVDs (1220, 99.6%). 5 products were sold as pre-filled cartridges or pre-filled EVDs. Where bottles of SCRA liquids were sold, the most common bottle sizes were 5mL (60%), 10mL (11%), or 7mL (4.5%), while18% did not have a bottle size listed. The most common total volume of SCRA liquid that could be purchased was 5mL, while the median was 50mL (IQR: 10mL − 200mL; range: 1mL − 20,000mL).

**Fig. 1:**
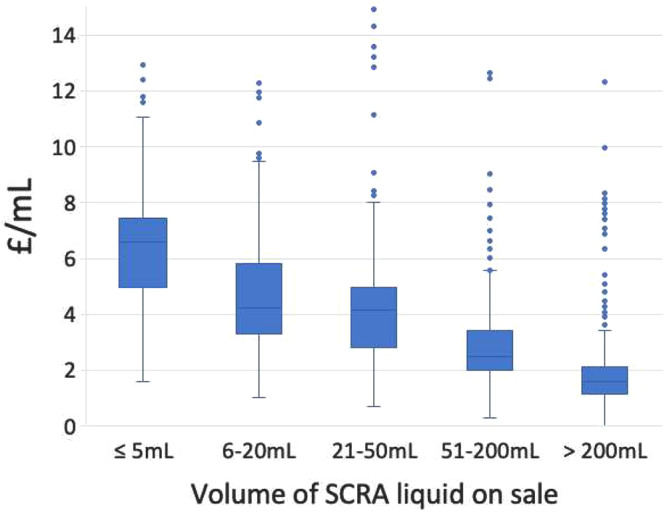
Box and whisker plot showing median, interquartile range, range and outliers of price per volume of SCRA liquid for different volume categories

The median price of liquid product was £3.39/mL (IQR: £2.01/mL - £5.29/mL). The majority of websites offered discounts with increasing volume of liquid purchased (Fig. 1), with median prices decreasing from £6.58/mL for ≤ 5mL purchased, to £4.23/mL for 6–20mL, £4.13/mL for 21–50mL, £2.48/mL for 51-200mL and £1.60/mL for > 200mL.

## Discussion

This study provides evidence of a rapidly evolving and active online marketplace from which SCRA vaping products seem to be easily obtainable, but also difficult to identify as controlled substances. Using simple search terms on a widely used search engine, we identified 62 websites selling SCRA vaping products, with 1225 individual listings and 128 unique brands. Although not a direct comparison, a survey of SCRA availability on the “dark web” in 2016 and 2017 which used different methodology to this study [15] found 32 individual SCRA branded vape products, which may suggest that this market has grown in the interim and/or that suppliers are increasingly marketing these on the surface web rather than the dark web.

There was evidence of both UK-based and international production and trade of these products. There was also evidence of companies competing for custom by offering incentives and discounts, including a wide variety of payment methods, measures to evade detection by law enforcement, and methods of ensuring anonymity in both payment and delivery.

As found in previous SCRA studies [31], products were heavily marketised, with colourful branding and abstract names that gave little or no hint of the contents of the product. Several SCRA vaping product brand names found in this survey have been listed in a previous survey of SCRA vaping products, such as “Bizarro”, “Diablo” and “Green Giant” [15], and in a survey of non-vape SCRA products, such as “K2” and “Mr Nice Guy” [31]. However, many previously common SCRA brand names were not found and many new brand names had appeared. This suggests a rapidly changing marketplace with ongoing product development and marketing.

SCRA vaping products were almost exclusively on sale as liquid formulations for refilling EVDs. It therefore does not appear that the rise in popularity of disposable nicotine e-cigarettes [17] has so far impacted on the online SCRA vaping product market. The most common bottle size on sale was 5mL. Given the lack of data on the concentration of SCRA in these liquids, it is difficult to quantify how many SCRA doses this may constitute. However, this volume of liquid is likely to provide at least several hundred puffs and, given the potency of known SCRAs, this is likely to represent several hundred psychoactive “doses”. For reference, since 2020 UK e-cigarette tanks are limited to a maximum volume of 2mL.

SCRA vaping products were offered both in small volumes consistent with supply for personal use, and large volumes suggestive of supply for further distribution. Prices varied significantly by brand, website and by the size of the purchase, however average prices by volume were similar to those found in a previous survey [15].

Of the minority (4.5%) of SCRA vaping products in which an active compound was listed, the most common was 5 F-AKB48. This fourth generation SCRA [32] was also a common compound found in a previous Europe-wide SCRA snapshot survey performed in 2017 [16] and in serum analyses of patients presenting with drug toxicity to an emergency department in London in 2015 [33]. Of the other SCRA compounds listed in this study, they were a combination of first generation (e.g. JWH-018) and later generation SCRA compounds. It is likely that 5 F-MDMB-PINACA and 5 F-ADB refer to the same compound as each other, as do 5 F-CUMYL-PINACA and SGT-25 [34]. Interestingly, there was no advertising of AB-CHMINACA or MDMB-CHMICA, two SCRA compounds which have become relatively well-known for being associated with significant toxicity [35–37].

Several factors raise concerns that SCRA vaping products could be purchased and consumed by undiscerning customers, or misused even by experienced consumers. Websites were easily accessed and products were available at a range of prices. Products were generally sold with minimal or no explanation of their contents or how to use them. Where descriptions of ingredients or psychoactive effects were provided, they tended to be abstract, vague or incorrect. There was generally no information on the chemical make-up of active ingredients or solvents, conditions of preservation, expiry date or EVD settings necessary for use. SCRA vaping products were often sold alongside legal products such as nicotine-containing EVDs or CBD products and, concerningly, many websites stated their products were legal.

We used standardised methodology developed by the EMCDDA which has been used in multiple studies in recent years [28, 29]. Nevertheless, the websites found were limited by the search methods and search terms chosen. Information on websites could not be confirmed, particularly as no purchases were made and no chemical analyses were performed; in fact, it is likely some information on websites was faked in order to evade identification and legal action. There is no way of knowing actual numbers of sales from these websites, especially given the anonymity afforded by their payment options– indeed, the authors of a previous snapshot study struggled to obtain some ordered samples [16]. Here, products clearly intended for vaping were studied, however it is noted that EVDs with correct settings can be used to inhale most drug forms including powders and herbs sprayed with active ingredient.

Further studies are needed, both to purchase and confirm the chemistry of these products, and to assess the pattern of their use and their clinical effects on end users. Nonetheless, the ease with which these products can be obtained has implications both for clinicians and policymakers,

Clinicians assessing patients experiencing the toxic effects of products purchased online should be aware of the availability of SCRAs and their potential use in EVDs. Thorough toxicological assessment should always include questions on inhalation of substances via vape, and the name of any vaped substances should be enquired about–product names identified in this study can be used for reference if SCRA use is suspected. Clinicians should be aware that patients may believe that the substances they have inhaled are legal.

Policymakers, both in the UK and elsewhere, should be aware that legislation making SCRA products illegal has not limited the expansion of an easily accessible online SCRA marketplace. The apparent international nature of trade in SCRA products suggests that international cooperation may be needed when exploring approaches to limiting their distribution, while the presence of SCRA products on the surface web suggests dialogue between policymakers and tech companies may also be important when exploring ways to reduce harm from these products. The ease of availability of NPS specifically for use with EVDs on the surface web is of relevance to policy decisions regarding vaping in general. Ongoing monitoring of the online market place, in conjunction with data from drug seizures, hospital presentations and other monitoring tools, is needed to maintain an understanding of evolving patterns of trade and use of SCRA vaping products, in order to inform future policy aimed at limiting the potential harms caused by the sale and use of these substances.

## Data Availability

The data that support the findings of this study are available from the corresponding author, AG, upon reasonable request.

## Declarations

### Presentation at meetings

Data in this manuscript will be presented at the 44th Congress of European Association of Poisons Centres and Clinical Toxicologists, Munich, Germany, 2024.
